# ABSI is a poor predictor of insulin resistance in Chinese adults and elderly without diabetes

**DOI:** 10.20945/2359-3997000000072

**Published:** 2018-10-01

**Authors:** Kai Wu, Sen He, Yi Zheng, Xiaoping Chen

**Affiliations:** 1 Sichuan University Sichuan University West China Hospital Department of Cardiovascular Medicine Chengdu China Department of Cardiovascular Medicine, West China Hospital, Sichuan University, Chengdu, China; 2 Guangyuan Central Hospital Department of Cardiovascular Medicine Guangyuan China Department of Cardiovascular Medicine, Guangyuan Central Hospital, Guangyuan, China; 3 903 Hospital Department of Cardiovascular Medicine China Department of Cardiovascular Medicine, 903 Hospital, and Center for Medical Radiation Biology, Institute of Materials, China Academy of Engineering Physics, Mianyang, China; China Academy of Engineering Physics Institute of Materials Center for Medical Radiation Biology Mianyang China

**Keywords:** Body shape index, body mass index, waist circumstance, insulin resistance

## Abstract

**Objective:**

Recently, a new obesity index (A Body Shape Index, ABSI) based on waist circumference (WC) was developed, and high ABSI corresponds to a more central concentration of body volume. It is well known that central obesity is closely linked with insulin resistance (IR). Therefore, our study aimed to examine the discriminatory power of ABSI for IR in Chinese adults and elderly without diabetes.

**Subjects and methods:**

In 2007, a cross-sectional study was made. In this study, 570 individuals without diabetes were available for analysis (male: 56.1%, mean age: 62.3 ± 6.5 years). Insulin resistance was assessed by homeostasis model assessment (HOMA-IR). Areas under the receiver operating characteristic (ROC) curves were determined to identify variables/models that could predict insulin resistance.

**Results:**

ABSI was associated with IR, the cut-off points was 0.0785 m11/6kg-2/3 to identifying IR and the area under the ROC (AUC) curve was 0.618 (95%CI: 0.561-0.675), which was not better than body mass index BMI (AUC = 0.753; 95%CI: 0.706-0.801), WC (AUC = 0.749; 95%CI: 0.700-0.797), and fasting plasma glucose (FPG, AUC = 0.752; 95%CI: 0.705-0.799). Furthermore, combination with ABSI could improve the discriminatory power of other variables for IR. The AUC curve increased from 0.753 to 0.771for BMI, 0.749 to 0.754 for WC, 0.752 to 0.769 for FPG, respectively.

**Conclusions:**

ABSI is associated with IR in the general Chinese adults and elderly without diabetes, but the discriminatory power for IR is poor. It is recommended that ABSI be used in combination with other variables.

## INTRODUCTION

Insulin resistance is characterized by a decrease in the ability of insulin to stimulate the use of glucose by muscles and adipose tissues and to suppress hepatic glucose production and output (1). Insulin resistance plays a patho-physiological role in type 2 diabetes and metabolic disorders, and is frequently present in hypertension, coronary artery disease, cancer, endothelial dysfunction and depression (2-6). Early identification of insulin resistant individuals is important for managing the health problems associated with insulin resistance.

It is well known that obesity is closely linked with insulin resistance (7), and many studies have shown that obesity indexes, such as body mass index (BMI) and waist circumstance (WC), could estimate insulin resistance (8-11). Recently, Krakauer and Krakauer (12) developed a new obesity index, namely a body shape index (ABSI), based on WC that is approximately independent of height, weight, and BMI. High ABSI indicates that WC is higher than expected for a given height and weight and corresponds to a more central concentration of body volume (12). Krakauer and cols. (12,13) have shown that ABSI could predict mortality in the general American and British population, even better than BMI and WC. In consideration of the aforementioned characteristics of ABSI and the present findings (12,13), we conclude that ABSI might potentially be a good marker of insulin resistance. Since ABSI was developed, it has led to substantial international interests (14-22). However, to the best of our knowledge, the specific relationship between ABSI and insulin resistance about Chinese adults and elderly was not studied previously, only some foreign studies were reported (23-25). Therefore, the aims of our study were to examine the discriminatory power of ABSI for insulin resistance in Chinese adults and elderly without diabetes.

## SUBJECTS AND METHODS

### Study population

In 2007, a cross-sectional study was conducted among 711 adults and elderly in an urban community located in Chengdu, Sichuan province, China. The study was supported by mega-projects of science research for the 11th five-year plan, China (Trends in the incidence of metabolic syndrome and integrated control in China). Among the 711 individuals, 141 of them had no data about insulin or were diagnosed with diabetes. Therefore, the remaining 570 individuals were available for analysis (male: 56.1%, mean age: 62.3 ± 6.5 years). The study was approved by Ministry of Health of China, as well as by the Ethics Committee of West China Hospital of Sichuan University. All participants gave informed consent.

### Data collection

Anthropometric measurements, such as height, weight and WC, were conducted at the time of interview. Height was measured using a digital stadiometer with a fixed vertical backboard and an adjustable head piece. Weight was measured on a digital scale. At the end of a normal exhalation, WC was measured to the midpoint between the lower border of the rib cage and the iliac crest. Blood samples were drawn from the antecubital vein in the morning after a 12-h fasting. Laboratory tests included fasting plasma glucose (FPG), low- density lipoprotein cholesterol (LDL-C), high-density lipoprotein cholesterol (HDL-C), triglyceride (TG) and insulin. FPG, LDL-C, HDL-C and TG were measured enzymatically using a MODULAR P800 Analyzer (Roche Diagnostics). Fasting serum insulin was measured by radioimmunoassay (XH-6010, Xi'an, China). These chemistries were measured at the laboratory of West China Hospital (Chengdu, China).

### Related definitions

Insulin resistance was assessed by homeostasis model assessment (HOMA-IR), which was calculated according to the following formula: fasting insulin (mU/mL) × fasting glucose (mmol/L)/22.5 (8). Insulin resistance was defined as being in the highest quintile of HOMA score (≥ 1.66), according to the previous studies (26,27). BMI was calculated as weight in kg/height in m^2^. ABSI was defined as WC/(BMI^2/3^height^1/2^), expressing WC and height in m (12). Those with hypertension were defined as having systolic blood pressure of at least 140 mmHg and/or diastolic blood pressure of at least 90 mmHg and/or currently taking antihypertensive medications. Diabetes mellitus was defined as one of the following at follow-up assessment: (1) fasting plasma glucose ≥ 7.0 mmol/L; (2) a positive response to the question, “Has a doctor ever told you that you have diabetes?”or (3) current use of insulin or oral hypoglycemic agents (14).

### Statistical analysis

Descriptive statistics (mean ± standard deviation, median + inter-quartile, percentages, etc.) were used to summarize demographic and metabolic characteristics. Correlations for normally distributed and skewed variables were assessed, respectively, by Pearson and Spearman correlation analysis. Krakauer and cols. (12) thought that ABSI expressed the excess mortality risk from high WC that was complementary to BMI and to other known risk factors. We also estimated whether combination with ABSI could improve the discriminatory power of other variables for insulin resistance. To estimate whether combination with ABSI could improve the discriminatory power of other variables for insulin resistance, different logistic regression models were developed. Areas under the receiver operating characteristic (ROC) curves were used to estimate the discriminatory power of each variable/model for insulin resistance. We use Hosmer and Lemeshow test to estimated whether combination with ABSI could improve the discriminatory power of other variables for insulin resistance. The statistic follows the chi-square distribution and a larger p value indicates the model fit better. In view of the influence of age and sex as variables, we run the analyses with the adjustment of age and sex.

We also set the optimal cut-off point, which represents the optimal combination of sensitivity and specificity for the study sample. For statistical analysis, SPSS software was used (version 17.0; SPSS, Chicago, IL), and statistical significance was defined as p < 0.05.

## RESULTS

### Demographic and metabolic characteristics

The demographic and metabolic characteristics of the 570 subjects (male: 56.1%, mean total age: 62.3 ± 6.5 years, mean male age: 63.3 ± 6.2 years, mean female age:61.0 ± 6.7) are provided in Table 1 according to gender. The mean total values were 23.5 ± 3.2 kg/m^2^, 82.0 ± 9.6 cm, 0.0786 ± 0.0047 m11/6kg-2/3 and 4.6 ± 0.7 mmol/L for BMI, WC, ABSI and FPG respectively, the mean male values were 23.4 ± 3.0 kg/m^2^, 83.6 ± 9.3 cm, 0.0792 ± 0.0046m^11/6^kg^−2/3^ and 4.6 ± 0.8 mmol/L, and the mean female values were 23.6 ± 3.4 kg/m^2^, 80.0 ± 9.7 cm, 0.0778 ± 0.0048m^11/6^kg^−2/3^ and 4.6 ± 0.7 mmol/L. Other variables are shown in Table 1. For age, SBP, DBP, HDL-C, insulin, HOMA, height, weight, and ABSI, there is statistically significant difference between male and female, and all the P values were less than 0.001. But for the other variables, there is no significant difference between male and female. Logistic regression analysis showed that ABSI, BMI, WC and FPG were independently associated with insulin resistance, with adjusted OR values 2.395 (95%CI: 1.358-4.225, p < 0.003), 1.357 (95%CI: 1.232-1.496, p < 0.001), 1.117 (95%CI: 1.081-1.154, p < 0.001) and 3.853 (95%CI: 2.557-5.807, p < 0.001) respectively. Correlation coefficients of ABSI with WC, BMI, height and weight were 0.621 (p < 0.001), 0.121 (p = 0.004), 0.168 (p < 0.001) and 0.180 (p < 0.001), respectively.

**Table 1:** Demographic and metabolic characteristics

Variable	Total (570)	Male (320)	Female (250)
Age (years)	62.3 ± 6.5	63.3 ± 6.2	61.0 ± 6.7*
SBP (mmHg)	134.7 ± 19.1	136.7 ± 18.2	132.2 ± 19.9*
DBP (mmHg)	79.4 ± 10.1	80.6 ± 10.0	77.9 ± 10.0*
FPG (mmol/L)	4.6 ± 0.7	4.6 ± 0.8	4.6 ± 0.7
TG (mmol/L)	1.5 (1.1, 2.2)	1.7 (1.1, 2.0)	1.7 (1.2, 2.2)
HDL-C (mmol/L)	1.4 (1.3, 1.7)	1.4 (1.2, 1.6)	1.6 (1.3, 1.8)*
LDL-C (mmol/L)	3.0 ± 0.7	3.0 ± 0.8	3.0 ± 0.7
Insulin (mU/L)	5.2 (3.6, 7.3)	4.8 (3.4, 6.6)	5.6 (4.1,8.1)*
HOMA	1.05 (0.72, 1.53)	0.98 (0.67, 0.98)	1.16 (0.84, 1.67)*
Height (cm)	162.2 ± 7.7	166.7 ± 5.9	156.6 ± 6.0*
Weight (kg)	62.0 ± 10.1	65.1 ± 9.4	58.0 ± 9.6*
BMI (kg/m^2^)	23.5 ± 3.2	23.4 ± 3.0	23.6 ± 3.4
WC (cm)	82.0 ± 9.6	83.6 ± 9.3	80.0 ± 9.7
ABSI (m^11/6^kg^−2/3^)	0.0786 ± 0.0047	0.0792 ± 0.0046	0.0778 ± 0.0048*
Hypertension (%)	50%	51.2	48.4

Data are presented as means ± SD, median (interquartile range), or percentage.

“*” means p < 0.05.

ABSI: a body shape index; BMI: body mass index; WC: waist circumference; SBP: systolic blood.

Areas under receiver operating characteristic curves for potential variables/models identifying insulin resistance

**Figure 1:**
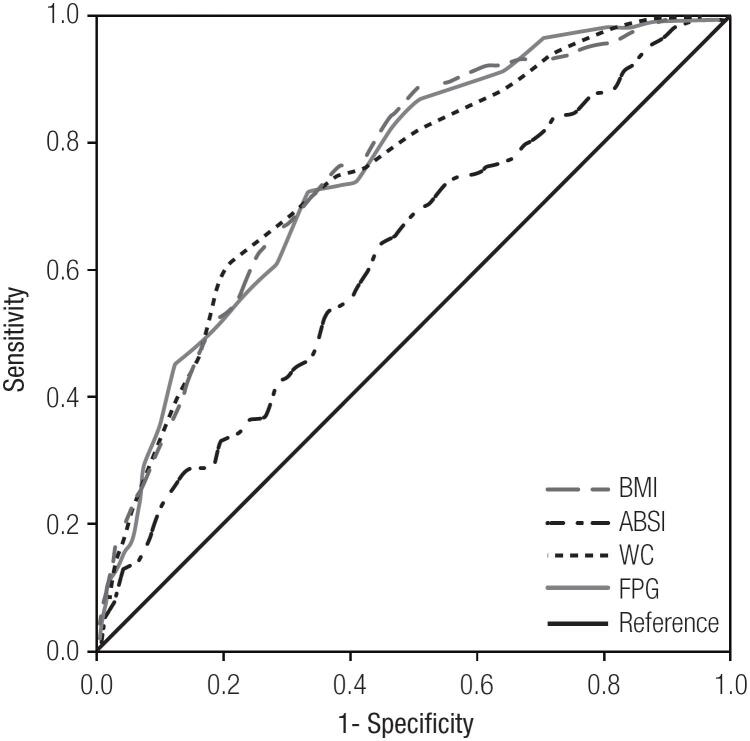
Receiver operating characteristic curves of BMI, WC, ABSI and FPG identifying insulin resistance. Abbreviations as in Table 1.

Table 2 presents the areas under the ROC curves identifying insulin resistance from potential variables. Among the anthropometric variables (BMI, WC, ABSI), the ROC curve analyses showed that ABSI had the poorest discriminatory power for insulin resistance, with an area under the ROC curve of 0.618 (95%CI: 0.561-0.675) (Table 2, Figure 1). Compared with ABSI, BMI and WC had better discriminatory power, as they had areas under the ROC curves of 0.753 (95%CI: 0.706-0.801) and 0.749 (95%CI: 0.7000.797), respectively (Table 2, Figure 1). The areas under the ROC curves of other variables were less than 0.700, except FPG (Table 2).

**Table 2:** Comparison of areas under receiver operating characteristic curves for potential variables identifying insulin resistance

Variable		Area under ROC Curve	95% CI
Biological variable			
	FPG	0.752	0.705 – 0.799
	TG	0.642	0.585 – 0.699
	HDL-C	0.351	0.296 – 0.405
	LDL-C	0.526	0.467 – 0.585
Anthropometric variable			
	BMI	0.753	0.706 – 0.801
	WC	0.749	0.700 – 0.797
	ABSI	0.618	0.561 – 0.675

ROC: receiver operating characteristic; CI: confidence interval. Other abbreviations as in Table 1.

We also estimated whether combination with ABSI could improve the discriminatory power of other variables for insulin resistance (Table 3). In combination with ABSI, BMI could improve the discriminatory power, and the area under the ROC curve increased from 0.753 to 0.771 (Tables 2 and 3). In combination with ABSI, WC and FPG could also improve the discriminatory power, and the areas under the ROC curves increased from 0.749 for WC to 0.754 in model 2, from 0.752 for FPG to 0.769 in model 3, respectively (Tables 2 and 3).

**Table 3:** Comparison of areas under receiver operating characteristic curves for different models identifying insulin resistance

Variable	Model 1	Model 2	Model 3
ABSI	√	√	√
BMI	√		
WC		√	
FPG			√
Model formula	-16.029+89.151*ABSI+0.312*BMI		-14.626+85.819*ABSI+1.343*FPG
Hosmer and Lemeshow test (p value)	0.133	0.302	0.091
AROC (95%CI)	0.771 (0.725 – 0.818)	0.754 (0.706 – 0.802)	0.769 (0.723 – 0.816)

For each model, ABSI in m^11/6^kg^−2/3^, TG in kg/m^2^, WC in cm and FPG in mmol/L. The symbol “√” meant that the specific variable was included in the specific model. Abbreviations as in Tables 1 and 2.

When considering age and sex as variables of adjustment in the analyses, we found that both age and sex affect the results as to BMI and WC, but as to ABSI and FPG, only sex alone affect the result. As to the ROC of different models, ABSI ROC curve analysis according to the sex, the male area under the curve is 0.689, female is 0.599, the difference is very big still.

The optimal cut-off points to identifying insulin resistance in our population were the following values: 24.2 kg/m^2^ for BMI, 87.5 cm for WC, 0.0785 m11/6kg-2/3 for ABSI, 4.75 mmol/L for FPG, −1.26 for model 1, −1.31 for model 2 and −1.31 for model 3, respectively; and other parameters for these optimal cut-off points are shown in Table 4.

**Table 4:** Sensitivity and specificity of different variables/models for identifying insulin resistance

Variable/Model	Optimal cut-off point	Sensitivity (%)	Specificity (%)	+ LR	-LR
BMI	24.2	72.2	66.8	2.17	0.42
WC	87.5	60.0	80.2	3.03	0.50
ABSI	0.0785	65.2	54.3	1.43	0.64
FPG	4.75	72.2	66.8	2.17	0.42
Model 1	-1.26	73.0	74.1	2.82	0.36
Model 2	-1.31	71.3	71.4	2.49	0.40
Model 3	-1.31	71.3	71.4	2.49	0.40

+LR: positive likelihood ratio; -LR: negative likelihood ratio. Other abbreviations as in Table 1.

## DISCUSSION

Recently, ABSI was studied extensively, some studies declared it can predicted mortality of general people or affected incidence of obesity or metabolic disorder (3,7,12,23). The aims of our study were to examine the discriminatory power of ABSI for insulin resistance in Chinese adults and elderly without diabetes. Our findings showed that ABSI is associated with insulin resistance in the general Chinese adults and elderly without diabetes, but the discriminatory power for insulin resistance is poor. Furthermore, combination with ABSI could improve the discriminatory power of other variables for IR.

Insulin resistance is associated with many health problems, such as type 2 diabetes, metabolic disorders, hypertension, coronary artery disease, cancer, endothelial dysfunction, depression and pulmonary arterial hypertension (2-6,28). As to Asian, McKeigue and cols. (29) found that insulin resistance syndrome is prevalent in South Asian populations and associated with a pronounced tendency to central obesity. It can lead to a higher prevalence of diabetes (19% vs 4%), higher blood pressures, higher fasting and post-glucose serum insulin concentrations, higher plasma triglyceride, and lower HDL cholesterol concentrations. Lee and cols. (30) also found HOMA-IR could identify dysglycemia and type 2 diabetes mellitus. The direct relation between insulin resistance and fatness is well known (3,7). Banerji and cols. (31) also found that increased visceral fat was related to dyslipidemia and increased frequency of insulin resistance and may account for the increased prevalence of diabetes mellitus and cardiovascular disease in Asian Indians. The underlying mechanisms of obesity inducing insulin resistance include inflammation, mitochondrial dysfunction, hyperinsulinemia and lipotoxicity, oxidative stress, genetic background, aging, fatty liver, hypoxia and lipodystrophy (7,32). Many studies have shown that obesity indices, such as BMI and WC, could estimate insulin resistance (8-11), our findings confirmed these studies.

The new obesity indice, namely ABSI, is based on WC that is approximately independent of height, weight, and BMI (12). The developers of ABSI have claimed that the index is more related to visceral than peripheral fat, which indicates that ABSI might potentially be a good marker of insulin resistance. However, the present findings are opposite to the hypothesis. Currently, there is not sufficient information available for us to understand why, however, some speculations could be made. ABSI is based on an American population from NHANES 1999-2004 (mainly including Mexicans, blacks and whites) (12). The American study population of Krakauer and cols. (12) has a higher height, BMI and WC than our study population. On the other hand, Asian populations are more prone to abdominal obesity and low muscle mass compared with their Western counterparts (33-35). Therefore, the coefficients of ABSI based on American population might not be suitable for other populations, especially for Asian populations. For example, Haghighatdoost and cols. (15) concluded that ABSI was a weak predictor for CVD risks and metabolic syndrome among Iranian adults, and Maessen and cols. (16) suggested that ABSI was not capable to determine cardiovascular diseases presence in the Netherlandish population (16). The authors thought that body height might confound the values of ABSI for health problems (15,16). Cheung (18) showed that ABSI was less associated with incident hypertension than BMI and WC in the Indonesian population, which might be caused by the lower mean BMI and WC (18). Furthermore, the coefficients of ABSI might be influenced by the age, even if Krakauer and cols. (12) showed that ABSI correlation with mortality hazard held across the range of age. In an published article by us (14), the subjects were with a mean age of 48.1 ± 6.2 years, which were younger than the present population, and the correlation coefficients of ABSI with BMI, height and weight were 0.611 (p < 0.001), −0.040 (p = 0.292), 0.283 (p < 0.001) and 0.155 (p < 0.001), respectively. The correlation coefficients did not hold across the range of age. Those might imply the coefficients of ABSI might not be suitable for other populations. Although ABSI had poor discriminatory power for insulin resistance, it could improve the discriminatory power of other variables (Table 3).

The present study has some limitations that should be considered. Firstly, insulin resistance is traditionally determined by euglycaemic-hyperinsulinaemic clamp technique (36), but in general population, it is more convenient and cost effective to estimate it using HOMA IR, which is an established test in epidemiological studies (37,38). Secondly, insulin resistance based on HOMA IR has been defined differently in different studies. Values based on 50th percentile, 75th percentile, 90th percentile, lower boundary of the top quintile or tertile of HOMA score have been used previously (39). We defined insulin resistance arbitrarily as HOMA score greater than the 80th percentile (≥ 1.66), but this is commonly practiced (26,27). Thirdly, for the absence of an oral glucose tolerance test, some individuals with diabetes might be included in the analysis, which could confound the results to some extent. Fourthly, no comparisons between different races might be another limitation. In addition, some other study considered that refined estimations of HOMA-IR levels could not exclude the indices such as age and sex (40). As to nondiabetic Spanish, there are gender-specific differences in HOMA-IR, with increased levels in women over fifty years of age that may be related with changes in body fat distribution after menopause. So, it meant that age and sex might influence the result of HOMA-IR, but our mode design did not regard sex and age as influencing factors.

Of course, the choice of statistical method is very important, because different statistical method may result in discriminating results. In our research, logistic regression analysis revealed that these variables associated with IR, and ROC suggested the identified ability. They are the common methods of clinical research.

In conclusion, our findings showed that ABSI is associated with insulin resistance in the general Chinese adults and elderly without diabetes, but the discriminatory power for insulin resistance is poor. It is recommended that ABSI be used in combination with other variables. Further studies about ethnic specificities of ABSI are needed and warranted, as well as the complementary values of ABSI to other known risk factors.
